# Supplementary figures and images for: LysM receptors in Coffea arabica: Identification, characterization, and gene expression in response to Hemileia vastatrix
Source: PLoS One. 2022 Feb 10;17(2):e0258838. doi: 10.1371/journal.pone.0258838 (PMC8830669; doi:10.1371/journal.pone.0258838)

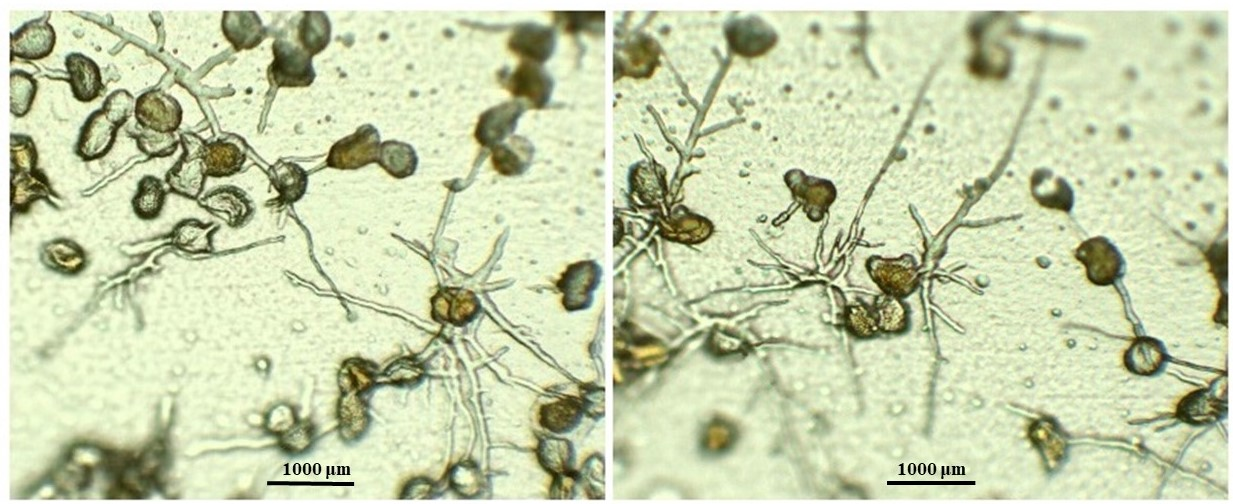

Supplement: S1 Fig — (TIF) [file pone.0258838.s001.tif]

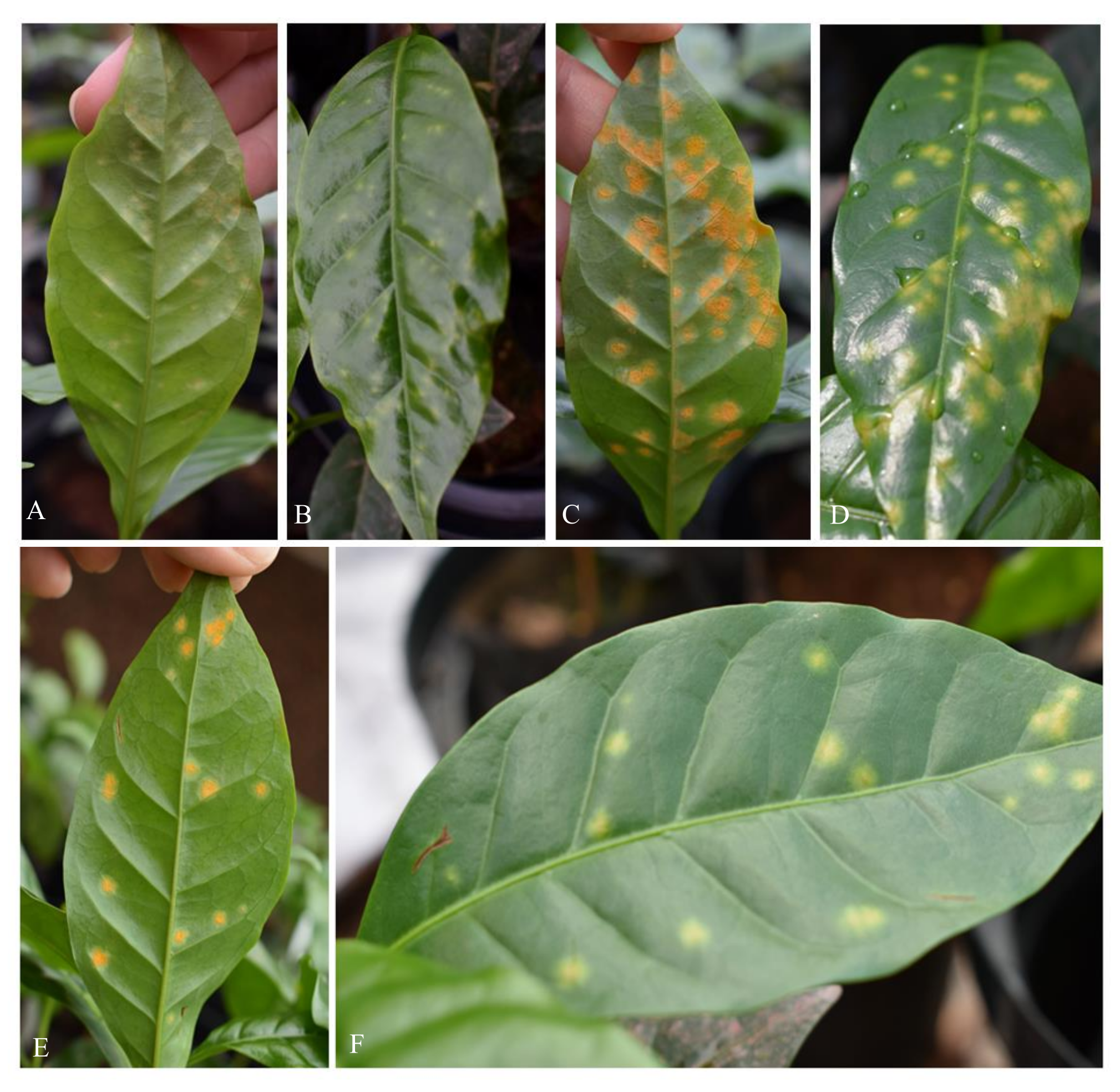

Supplement: S2 Fig — (A, B, C, D) Cultivar Mundo Novo IAC 367–4, (E, F) Catuaí Vermelho. (A) abaxial face 20 days after inoculation of the pathogen, (B) adaxial face 20 days after inoculation, (C, E) abaxial face 35 days after inoculation, (D) adaxial face 35 days after inoculation. (TIF) [file pone.0258838.s002.tif]

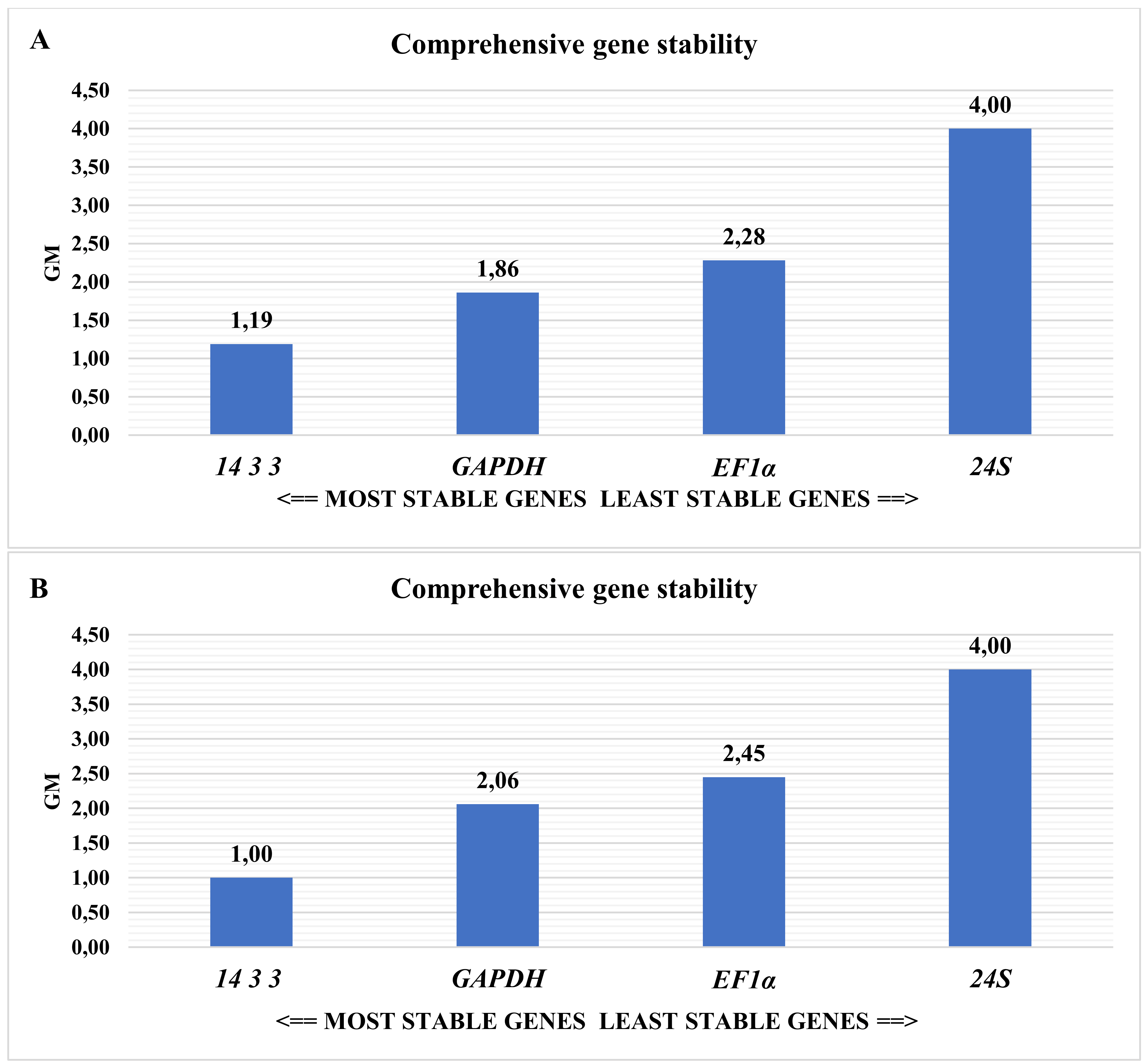

Supplement: S3 Fig — (A) Experiments 1, (B) Experiment 2. GM: Geometric mean of the weights from algorithms Delta-Ct, BestKeeper, NormFinder e geNorm. (TIF) [file pone.0258838.s003.tif]

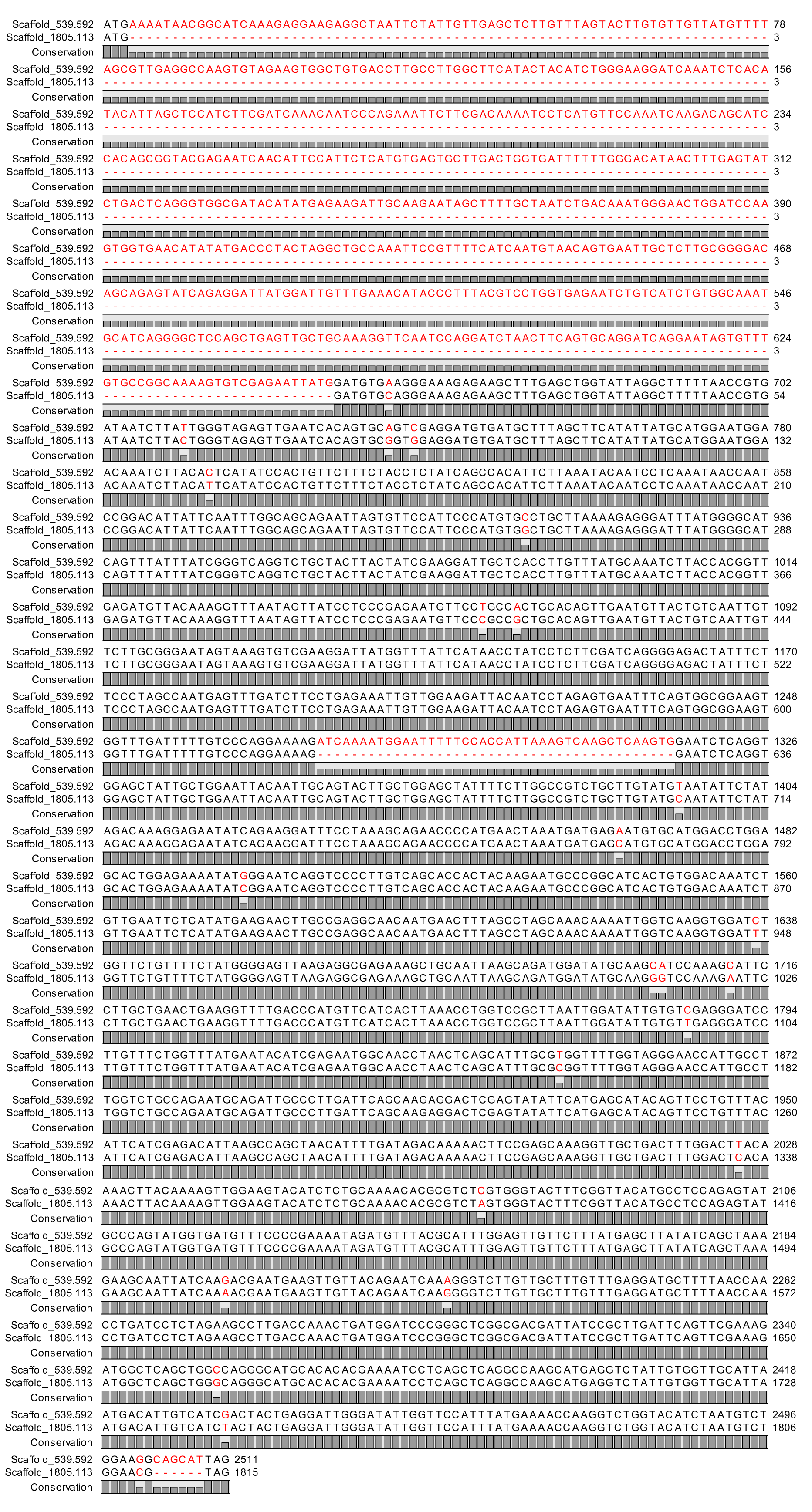

Supplement: S4 Fig — The alignments were obtained by CLC Genomics Workbench software. Gray bars show the conservation level of the positions; red letters, the different nucleotides; and red dashes, the gaps. Identity: 71, 33%. (TIF) [file pone.0258838.s004.tif]

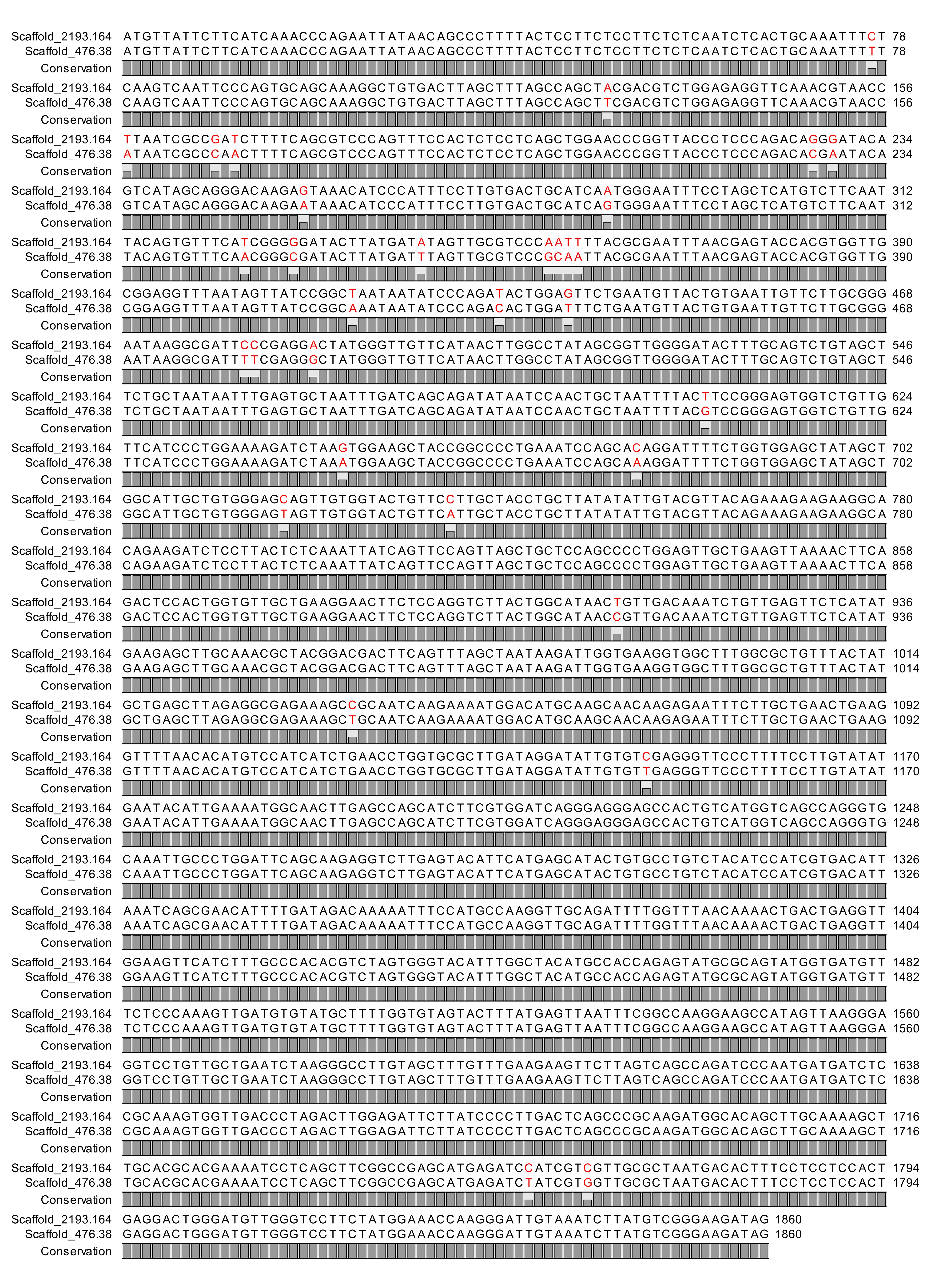

Supplement: S5 Fig — The alignments were obtained by CLC Genomics Workbench software. Gray bars show the conservation level of the positions; red letters, the different nucleotides; and red dashes, the gaps. Identity: 98,28%. (TIF) [file pone.0258838.s005.tif]

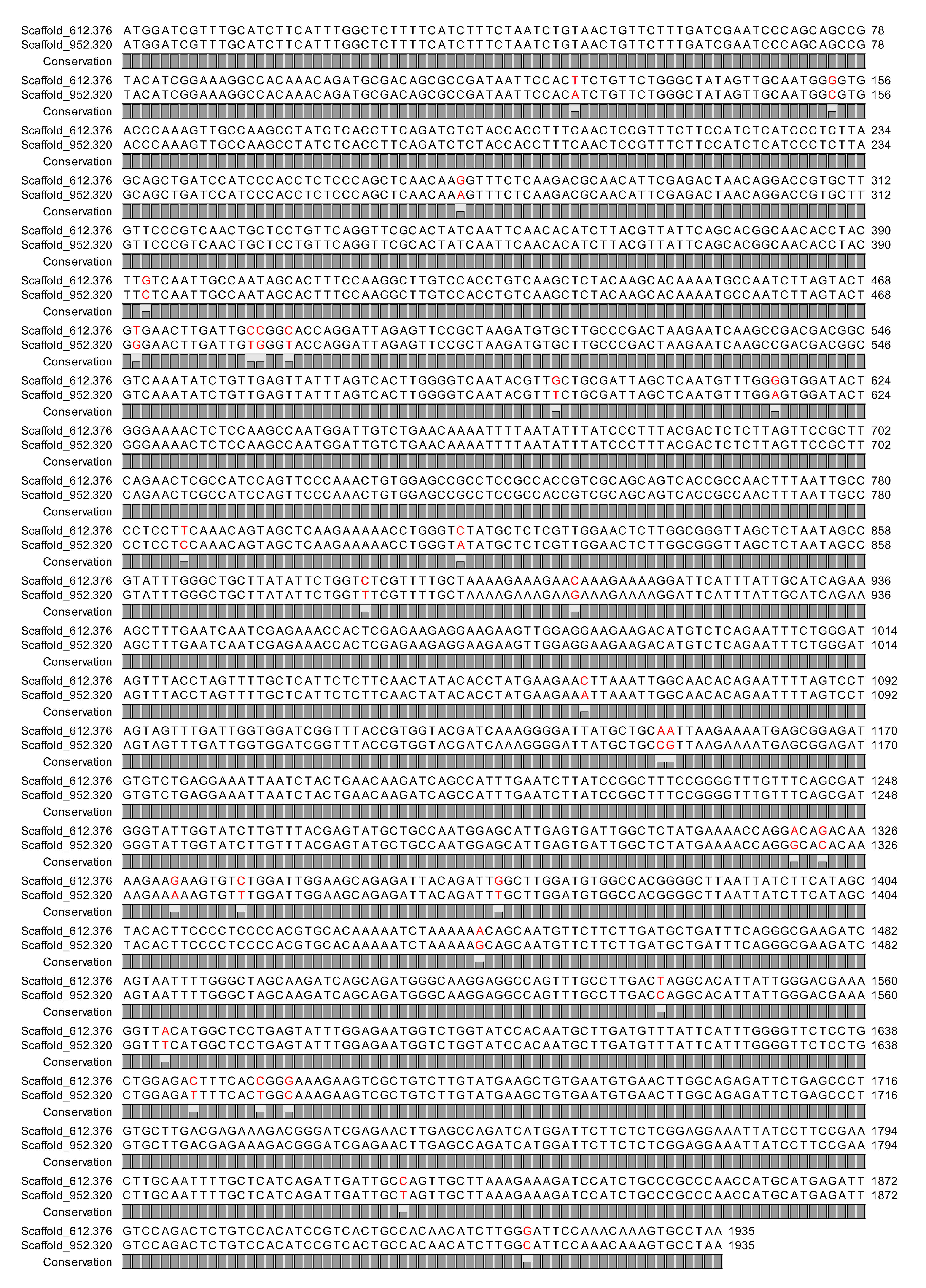

Supplement: S6 Fig — The alignments were obtained by CLC Genomics Workbench software. Gray bars show the conservation level of the positions; red letters, the different nucleotides; and red dashes, the gaps. Identity: 98,45%. (TIF) [file pone.0258838.s006.tif]
