# Supplementary material for: LysM receptors in Coffea arabica: Identification, characterization, and gene expression in response to Hemileia vastatrix
Source: PLoS One. 2022 Feb 10;17(2):e0258838. doi: 10.1371/journal.pone.0258838 (PMC8830669; doi:10.1371/journal.pone.0258838)
Supplement: S7 Fig — Each target gene is represented by the name: Ca1-CERK1, Ca2-CERK1, Ca1-LYP, Ca2-LYP, Ca1-LYK5, Ca2-LYK5, Ca-LYK4, 1433, GAPDH, EF1a and 24S. (PDF) [file pone.0258838.s007.pdf]

Ca1-CERK1

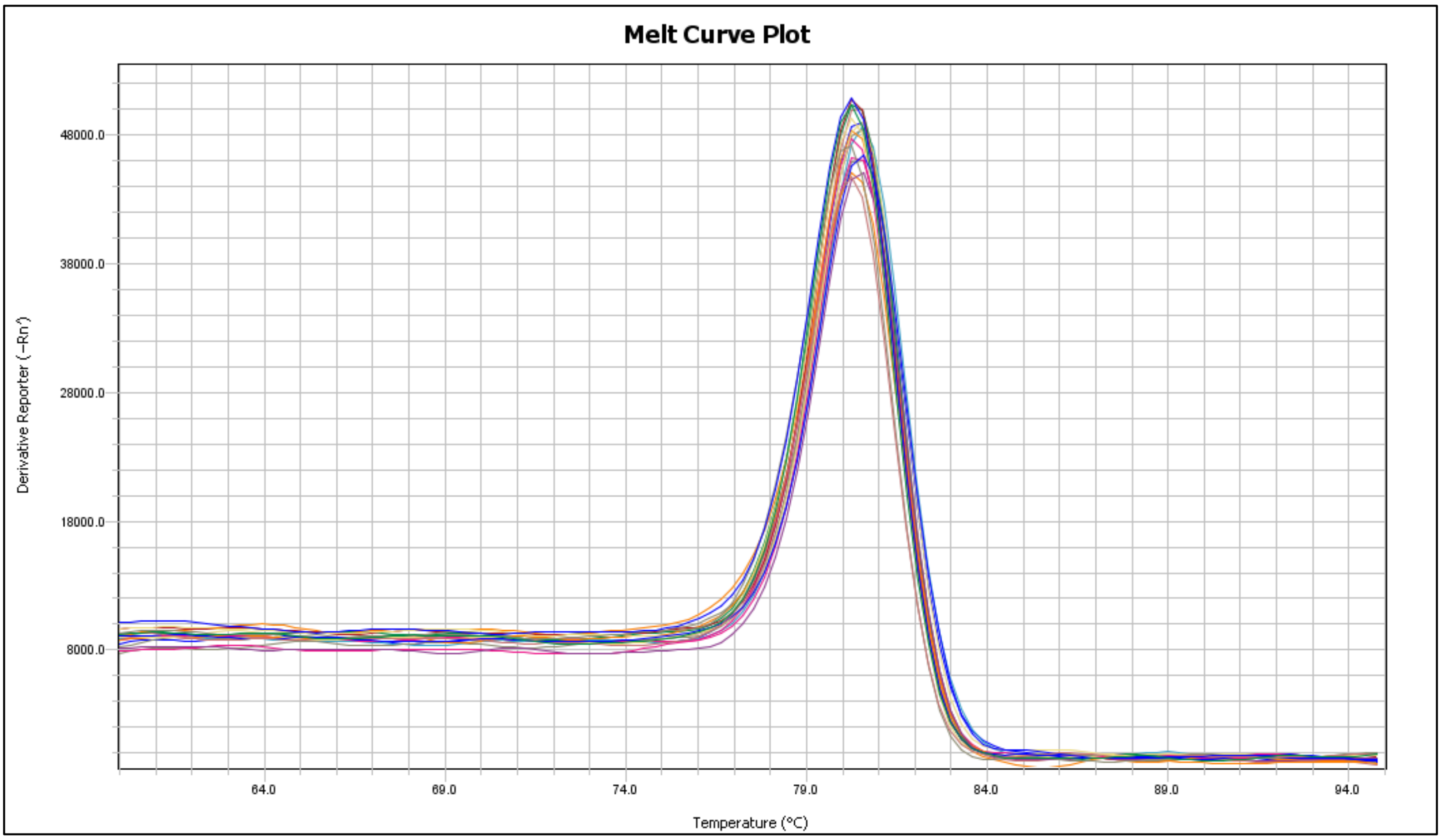

Ca2-CERK1

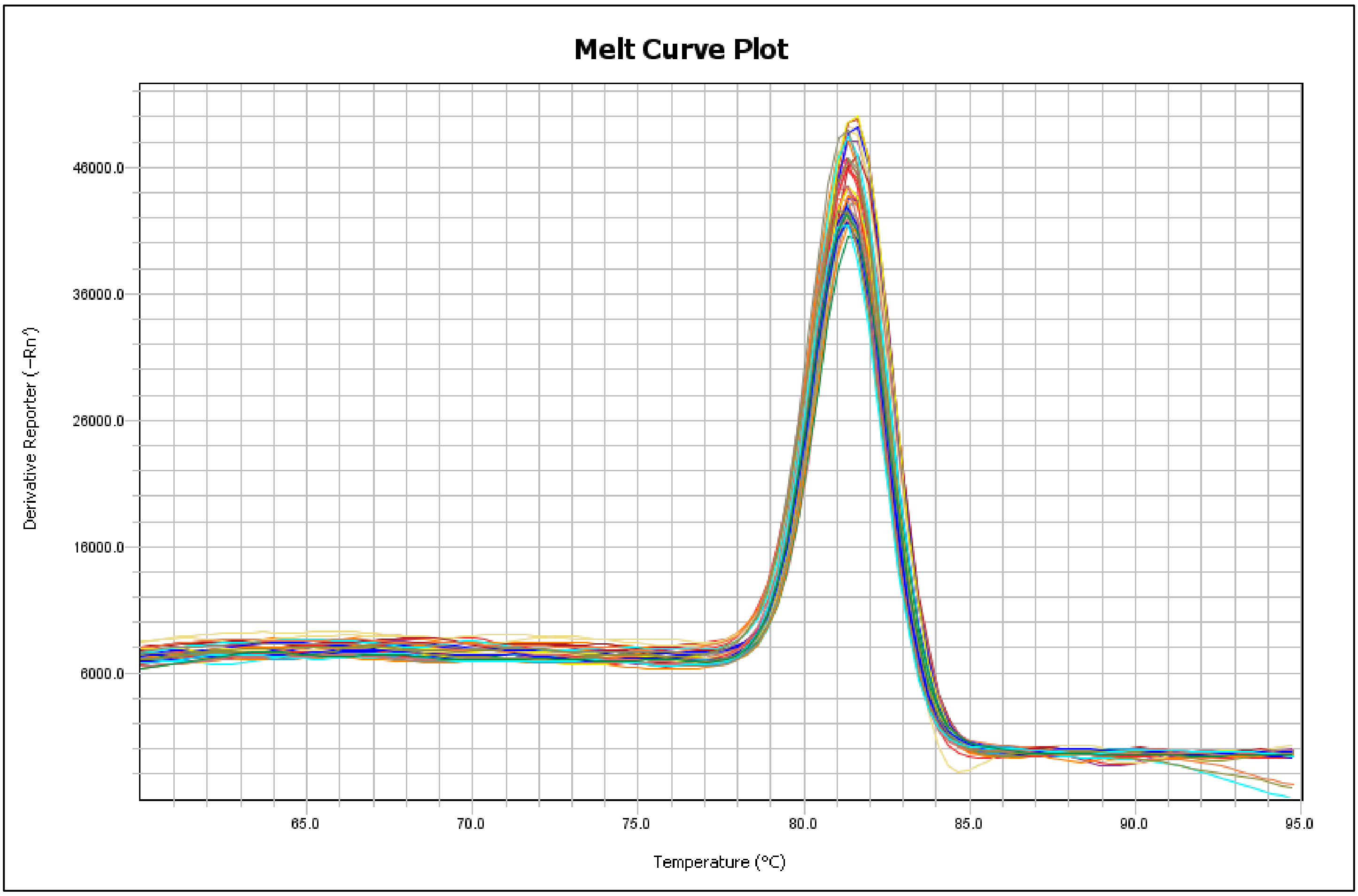

Ca1-LYP

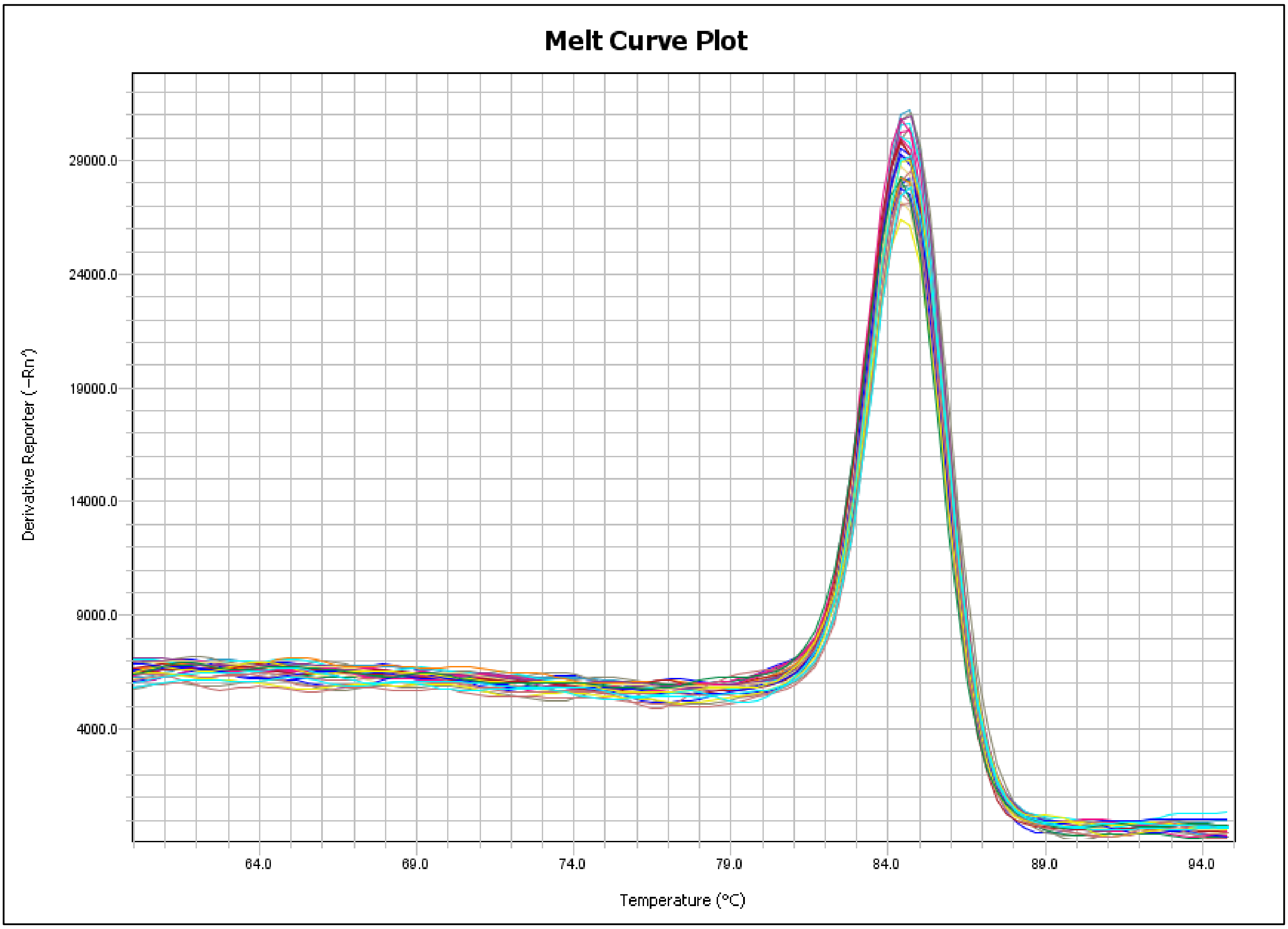

Ca2-LYP

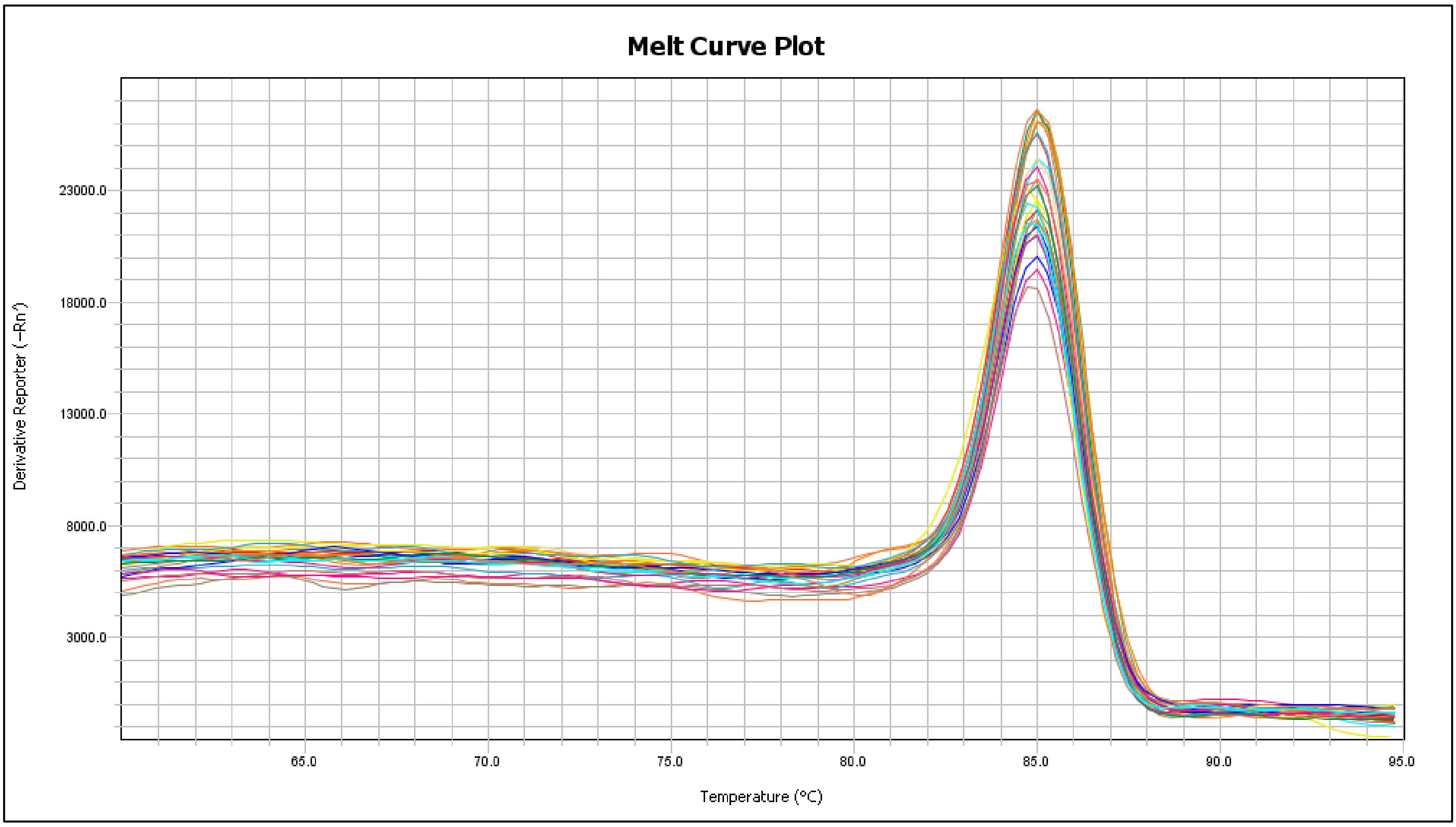

Ca1-LYK5

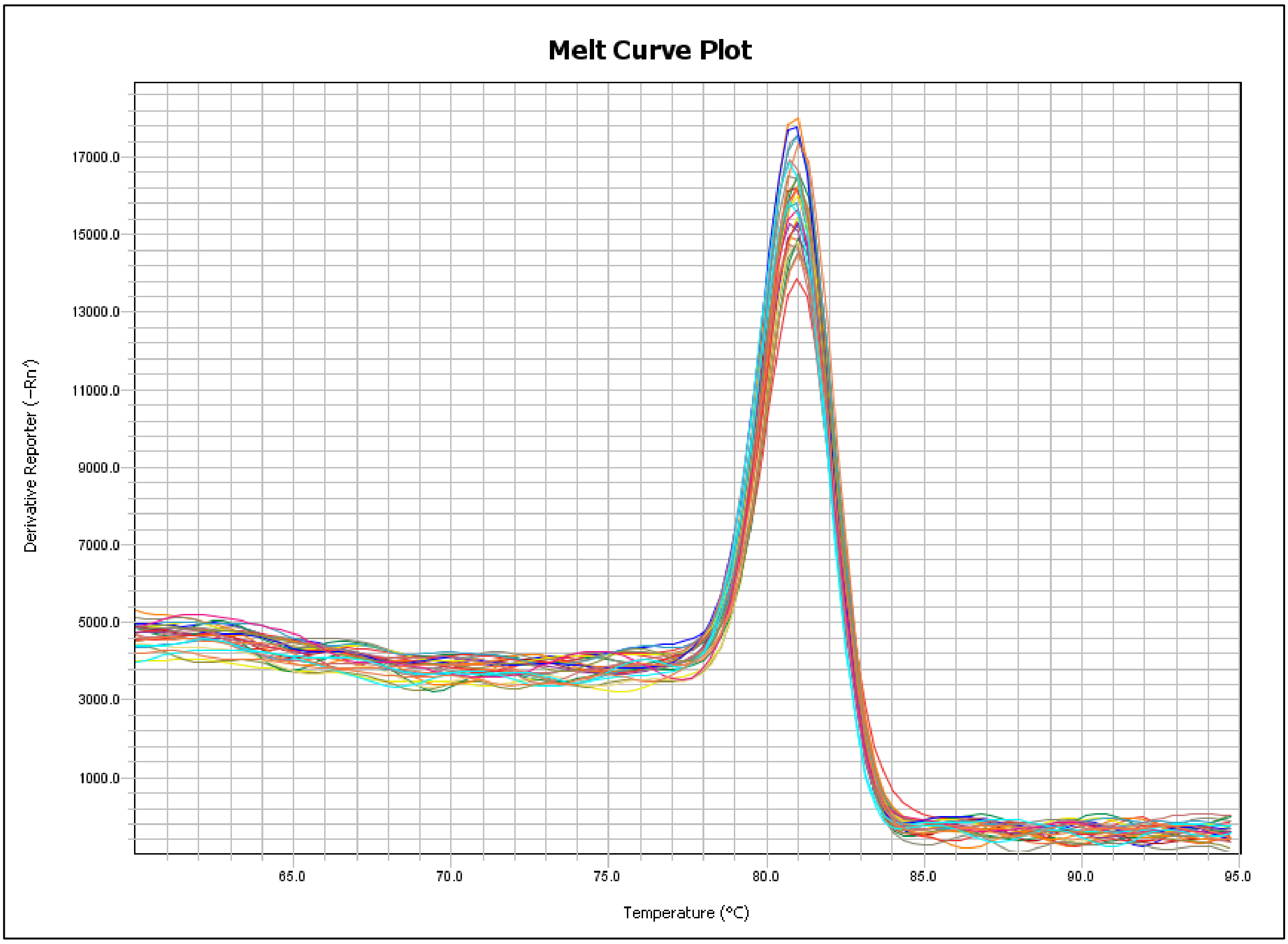

Ca2-LYK5

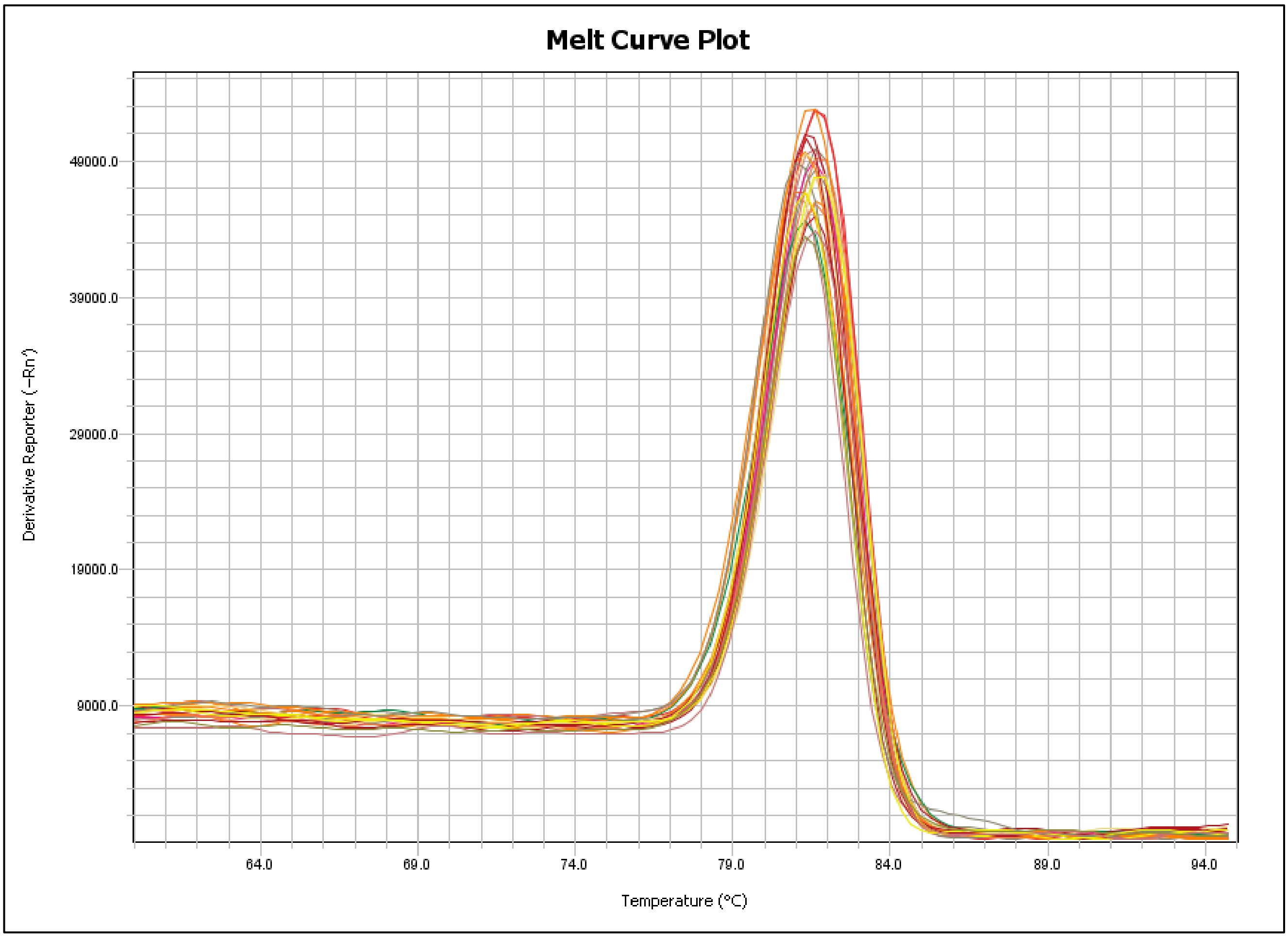

Ca-LYK4

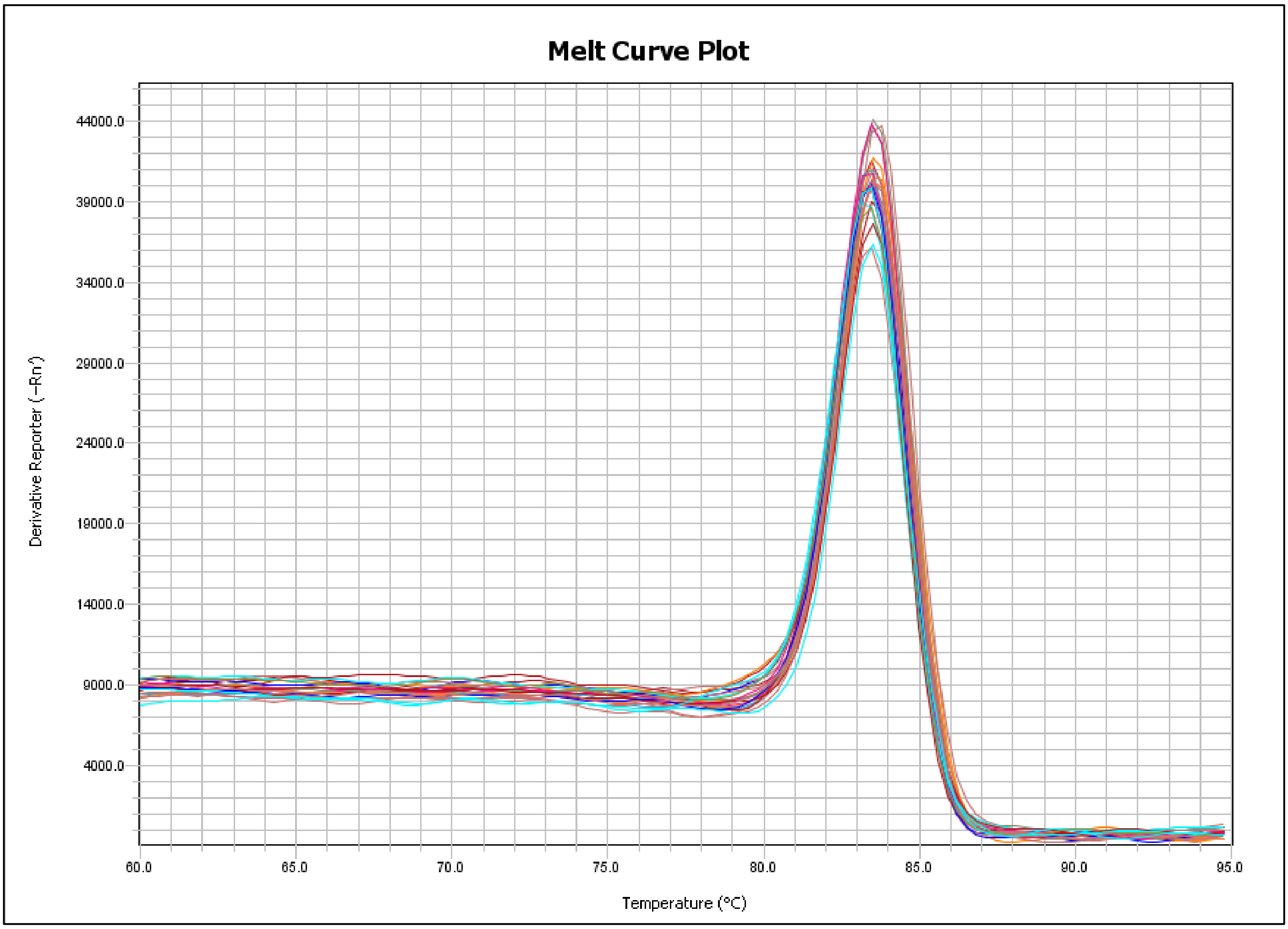
Melt Curve Plot
44000.0
39000.0
34000.0
29000.0
24000.0
19000.0
14000.0
9000.0
4000.0
Derivative Reporter (−Rn')
60.0
65.0
70.0
75.0
80.0
85.0
90.0
95.0
Temperature (°C)

1433

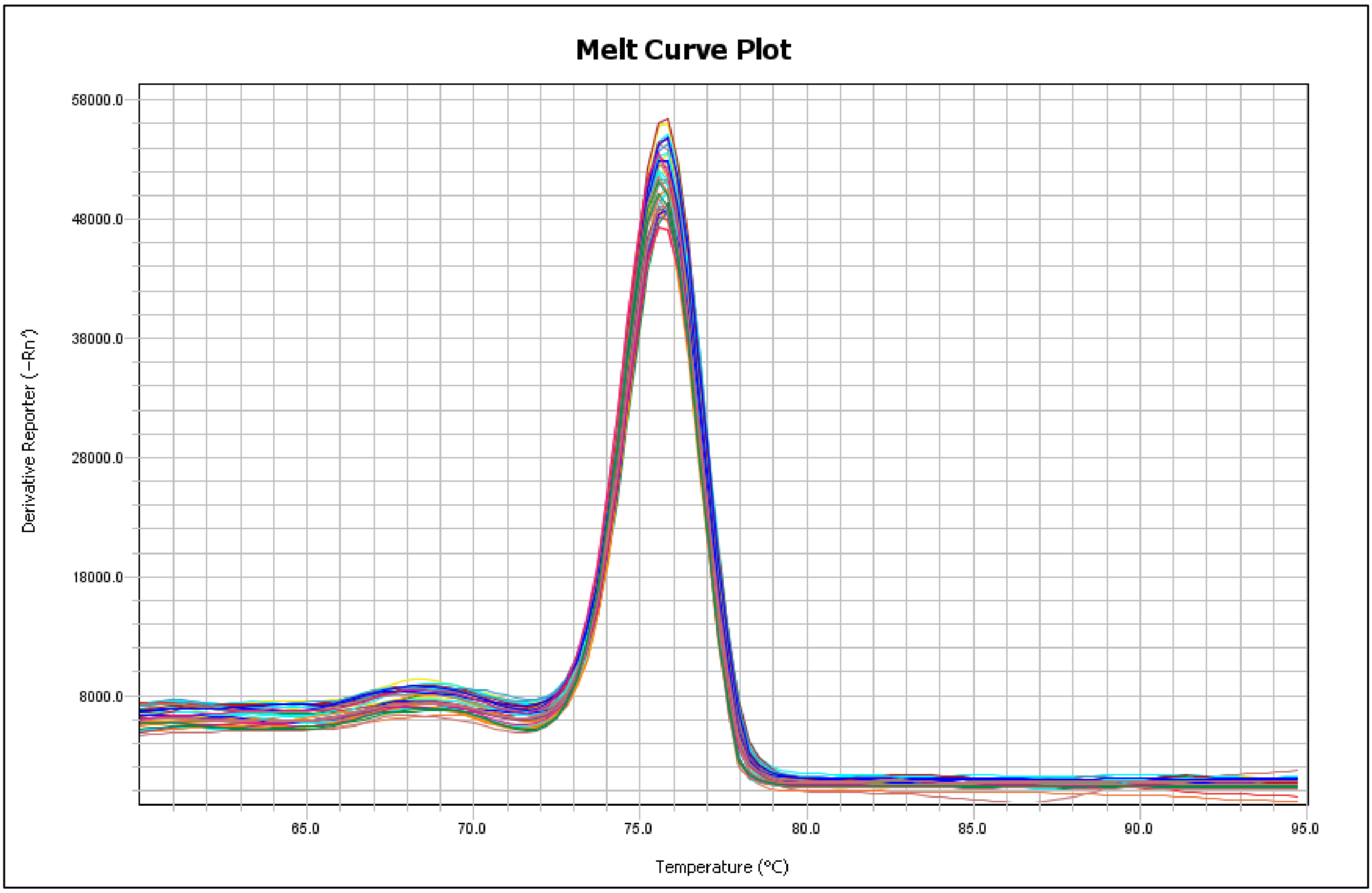
Melt Curve Plot
58000.0
48000.0
38000.0
28000.0
18000.0
8000.0
Derivative Reporter (−Rn')
65.0
70.0
75.0
80.0
85.0
90.0
95.0
Temperature (°C)

GAPDH

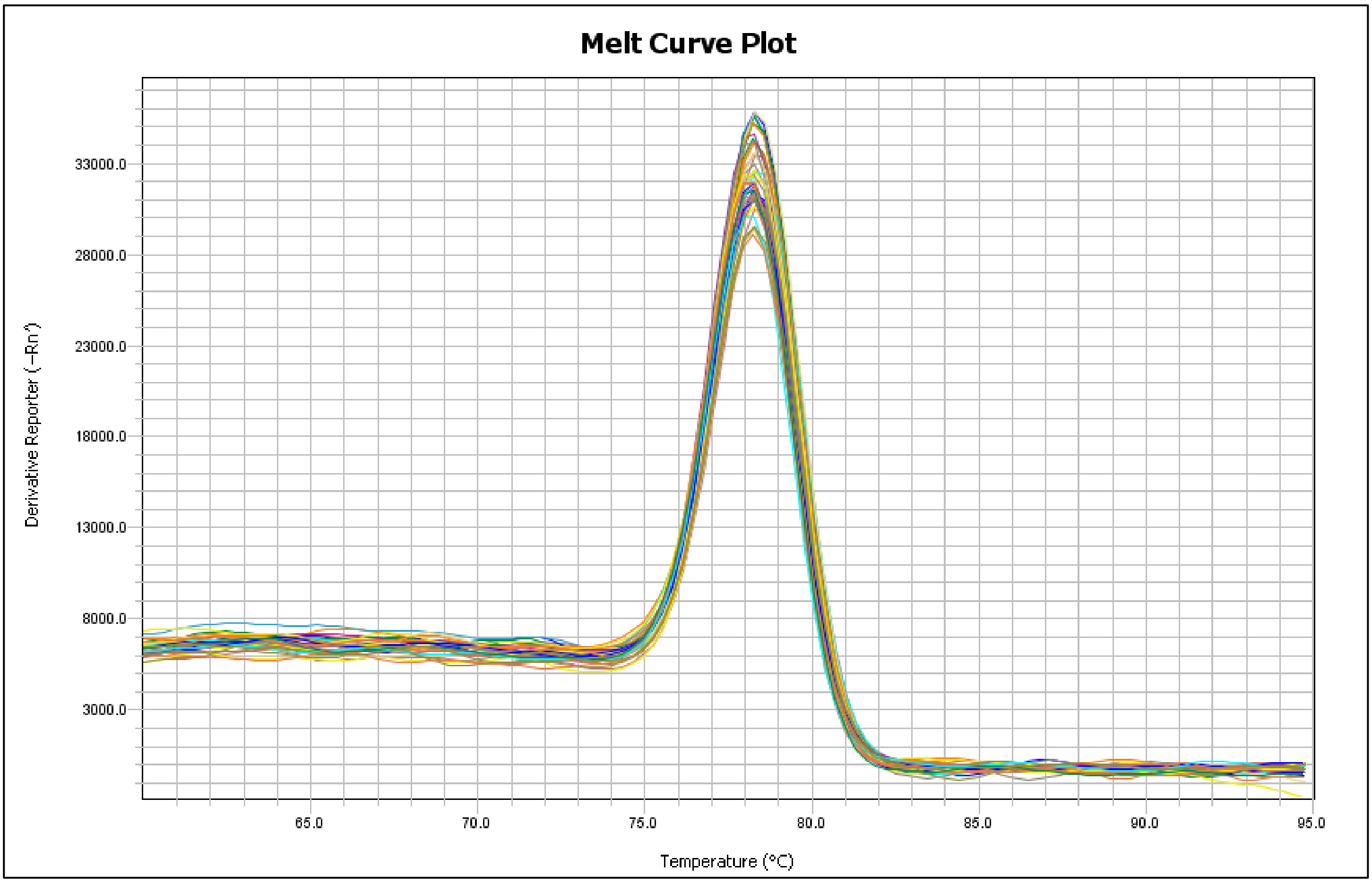

Melt Curve Plot
33000.0
28000.0
23000.0
18000.0
13000.0
8000.0
3000.0
Derivative Reporter (−Rn')
65.0
70.0
75.0
80.0
85.0
90.0
95.0
Temperature (°C)

EF1a

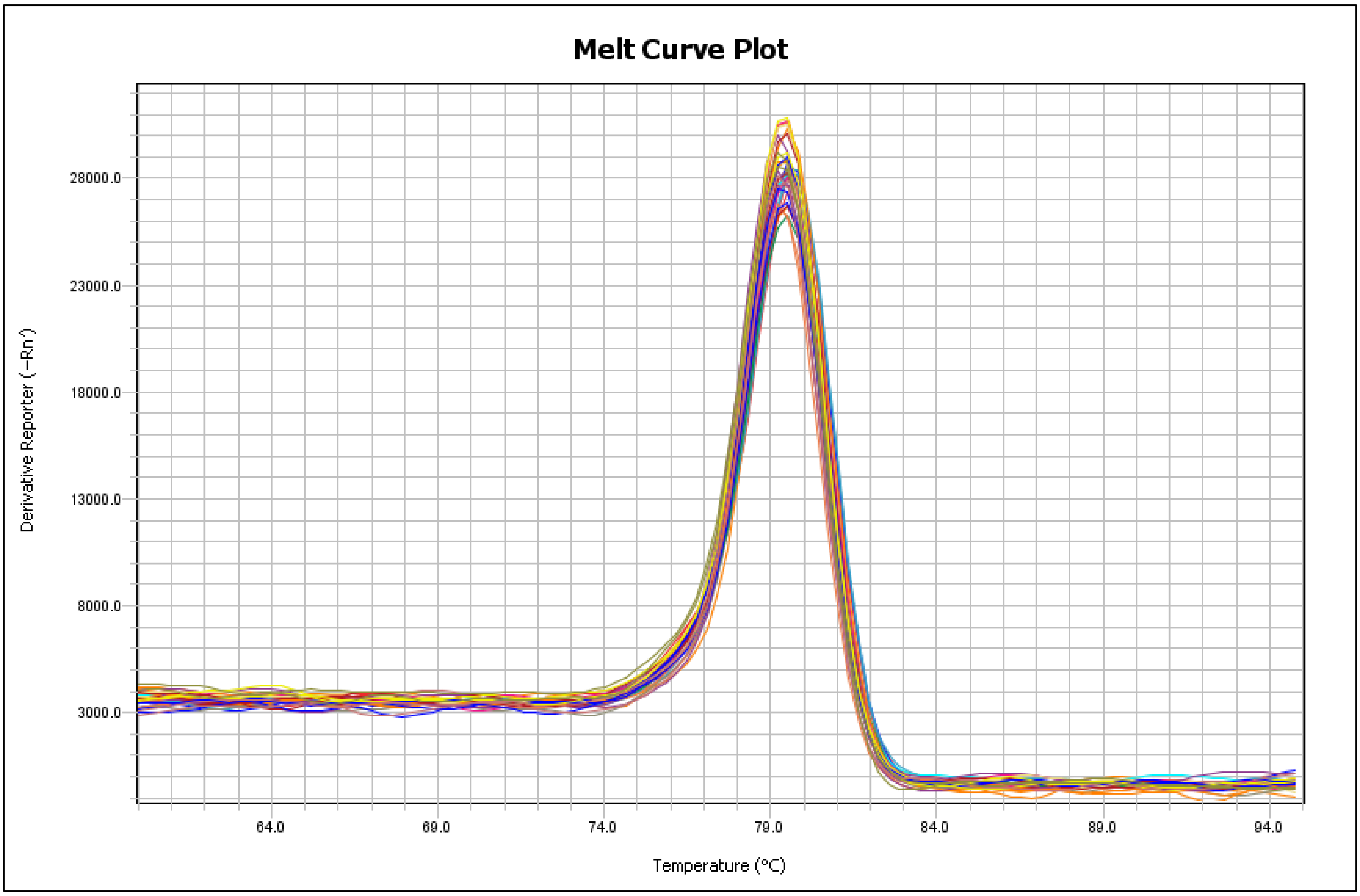

Melt Curve Plot
28000.0
23000.0
18000.0
13000.0
8000.0
3000.0
Derivative Reporter (−Rn')
64.0
69.0
74.0
79.0
84.0
89.0
94.0
Temperature (°C)

24S

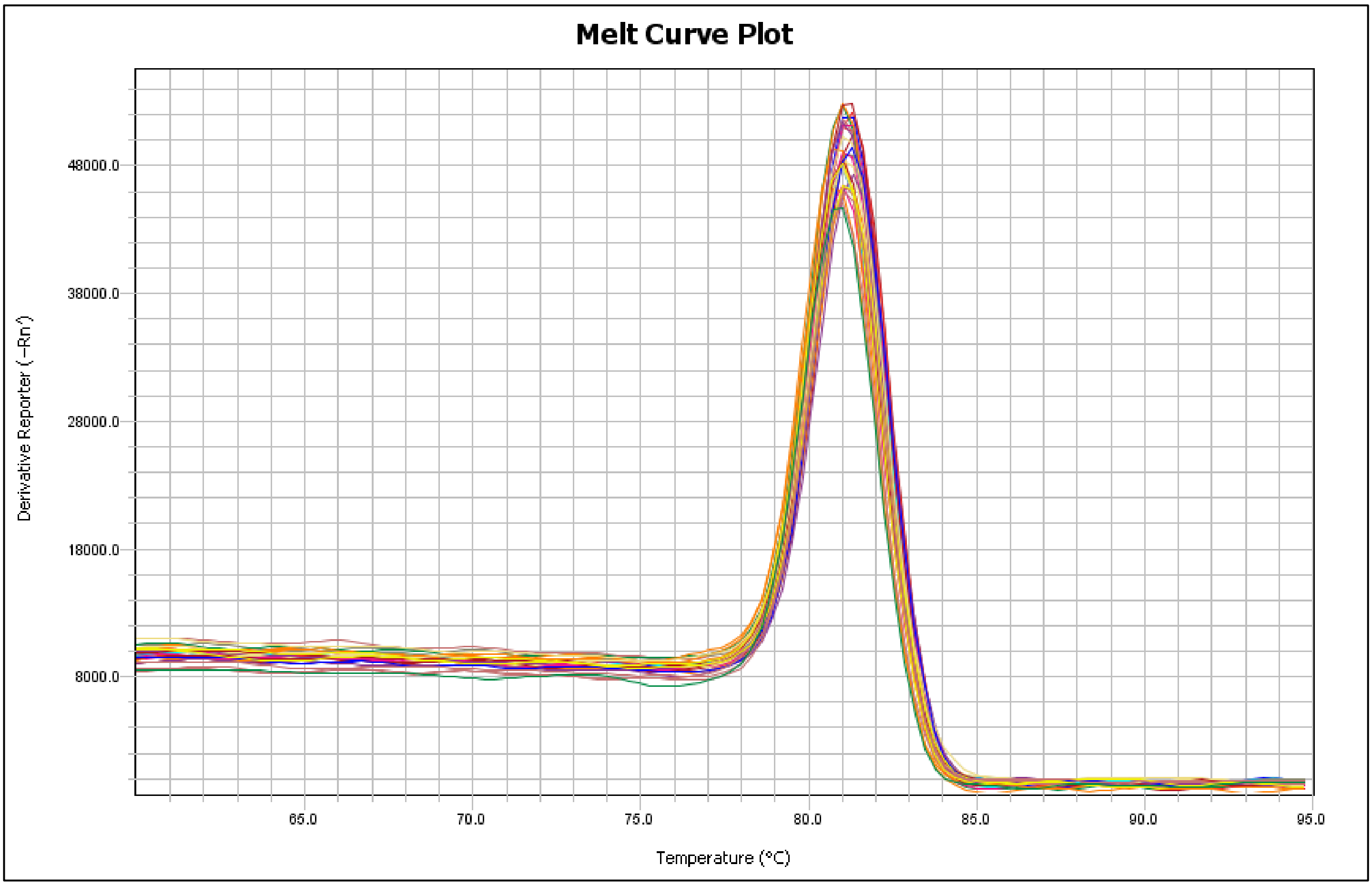

Melt Curve Plot
48000.0
38000.0
28000.0
18000.0
8000.0
Derivative Reporter (−Rn')
65.0
70.0
75.0
80.0
85.0
90.0
95.0
Temperature (°C)
